# Does miR-197 Represent a Valid Prognostic Biomarker in Head and Neck Squamous Cell Carcinoma (HNSCC)? A Systematic Review and Trial Sequential Analysis

**DOI:** 10.3390/jpm12091436

**Published:** 2022-08-31

**Authors:** Mario Dioguardi, Stefania Cantore, Diego Sovereto, Lucia La Femina, Francesca Spirito, Giorgia Apollonia Caloro, Marino Caroprese, Marta Maci, Salvatore Scacco, Lorenzo Lo Muzio, Michele Di Cosola, Giuseppe Troiano, Andrea Ballini

**Affiliations:** 1 Department of Clinical and Experimental Medicine, University of Foggia, Via Rovelli 50, 71122 Foggia, Italy; 2 Independent Researcher, 70129 Bari, Italy; 3 Unità Operativa Nefrologia e Dialisi, Presidio Ospedaliero Scorrano, ASL (Azienda Sanitaria Locale) Lecce, Via Giuseppina Delli Ponti, 73020 Scorrano, Italy; 4 Department of Basic Medical Sciences, Neurosciences and Sense Organs, University of Bari “Aldo Moro”, 70124 Bari, Italy; 5 Department of Precision Medicine, University of Campania “Luigi Vanvitelli”, 80138 Naples, Italy

**Keywords:** head and neck squamous cell carcinoma (HNSCC), noncoding RNA, oral cancer, miR-197, meta-analysis: Trial Sequential Analysis, bioinformatics analysis

## Abstract

(1) Background: Between tumors of the head and neck region, the squamous cell variant (HNSCC) is the most common and represents one of the main neoplasms affecting humans. At the base of carcinogenesis processes, there are genetic alterations whose regulation can be influenced by changes in the expression of microRNA (miR). Consequently, despite recent studies indicating miR-197 as a potential prognostic biomarker of survival for many varieties of cancer, there are currently no systematic reviews and trial sequential/bioinformatics/meta-analysis regarding the role of miR-197 in HNSCC. Our hypothesis was that with the existing literature, it is possible to clarify whether the different expressions of miR-197 in neoplastic tissues can represent a prognostic biomarker of survival in head and neck tumors. (2) Methods: The systematic review was reported following the indications of PRISMA and by consulting six electronic databases (including one register). Moreover, this review was carried out using the Kaplan–Meier plotter database portal, and hazard ratio (HR) data were extracted. Finally, a trial sequential analysis (TSA) was conducted to test the robustness of the proposed meta-analysis. (3) Results: This search identified 1119 articles and outcomes of the meta-analysis, reporting an aggregate HR for overall survival (OS) between the highest and lowest miR-197 expression of 1.01, 95% CI: [1.00, 1.02]. (4) Conclusions: We can state that, from the literature data included in the present meta-analysis, and from the TSA and bioinformatics analysis data, miR-197 does not currently represent a valid prognostic biomarker for HNSCC, although the data provided by the Kaplan–Meier plotter suggest that miR-197 can serve as a putative biomarker in short-term (5 years) survival.

## 1. Introduction

Head and neck squamous cell carcinoma (HNSCC) encompasses a heterogeneous group of squamous epithelial malignancies that are recognized as laryngeal squamous cell carcinoma (LSCC), oropharyngeal squamous cell carcinoma (OPSCC), hypopharyngeal squamous cell carcinoma (HSCC), and oral squamous cell carcinoma (OSCC), depending on the epithelium of origin [1].

At the base of the carcinogenesis processes, there are genetic alterations, whose regulation can be influenced by changes in the expression of certain genes and is associated with non-coding genes, such as MicroRNAs (miR) [2], a large group of small single-stranded non-coding endogenous RNAs, approximately 18–25 nucleotides in length, that play a significant role in the post-transcriptional regulation of genes through their interaction with 3′UTR of target m RNA.

Moreover, miRs are stable molecules that can be found not only in tissues but also in body fluids, such as blood, saliva, and urine [3,4,5]. In numerous studies, their dysregulation, which can also affect inflammatory and autoimmune processes related to carcinogenesis [6], has been associated with worsening survival prognosis for breast cancer [7], lung cancer [8], kidney cancer [6], osteosarcoma [3] and HNSCC [4].

Current evidence, even if still relatively limited, suggests that dysregulation of miR genes plays an important role in HNSCC. The downregulated miRs are miR-9, miR 125a, miR 136, miR-29c, miR-223, miR-187, miR 27, miR 34, miR 92, Let-7a, miR 124, miR139, miR 146a, miR 200, miR 145, miR-195, and miR 205. The upregulated miRs are: miR-375, miR-638, miR-200b-3p, miR-191-5p, miR-24-3p, miR-572, miR-1234, miR-103, miR-483-5p, miR-20a, miR-22, miR-29a, miR-17, miR-374b-5p, miR-425-5p, miR-122, miR-134, miR-184, miR 191, miR-412, miR-512, miR-8392, miR-21, miR-31, miR-155 miR-29b, mir-let-7c, miR-196a, and miR-196b [5,9].

According to previous research, the main miRs investigated that are related to a worsening of HNSCC prognosis are miR-21 [10], miR-155 [11], miR-31 [12], miR-196b [13], and miR-34a [14]. Furthermore, many other miRs are associated with a worse prognosis, and among these, we found that miR-197 revealed a fair prognostic potential in other neoplastic localizations.

Kumar et al. [15], found a significant overexpression of miR-197 in tumor specimens compared to normal lung tissues in the same patients. Other studies report that the deregulation of miR-197 is associated with a worsening of prognosis: Han and Liu [16], found a poor prognosis for gastric cancer, and similar results were reported for hepatocellular cancer in the study conducted by Zhan et al. [17]. Additionally, miR-197 appears to be downregulated in glioblastoma [18].

This non-coding RNA is located on chromosome 1p13.3 and has the stem–loop sequence GGCUGUGCCGGGUAGAGAGGGCAGUGGGAGGUAAGAGCUCUUCACCCUUCACCACCUUCUCCACCCAGCAUGGCC; mature miR-197-5p: CGGGUAGAGAGGGCAGUGGGAGG; mature miR-197-3p UUCACCACCUUCUCCACCCAGC.

Among the main data described in the literature, we found genes, signaling pathways and cascades such as Bcl-2, RAD51 [19], E-cadherin, p120-cadherin, FUS1 [20], Smad7, Net1A [21], c-Myc and cyclin D1 genes; signaling pathway EGFR-ERK1/2-MMP7; signaling pathway CKS1B/STAT3 [22]; signaling pathway ITGAV/STAT5 (prostate cancer) [23]; targeting in p53 (lung cancer) [24]; targeting LAMC2 and CD82 (gastric cancer) [25]; and KLF10/PTEN/PI3K/AKT cascade (bladder cancer) [26].

Accordingly, recent studies indicate miR-197 to be a potential prognostic biomarker of survival for many forms of cancer, but to date, no systematic reviews have been conducted on the role of miR-197 in HNSCC. Taking this context into account, our hypothesis was to clarify the prognostic potential of miR-197 as a survival biomarker for HNSCC in light of the new miR-197 studies currently available in the international scientific literature.

## 2. Materials and Methods

### 2.1. Protocol

The planning of this systematic review and meta-analysis was performed according to the recommendations of the Cochrane Handbook for Systematic Reviews of Interventions [27]. Our manuscript was prepared following the indications of the PRISMA (Preferred Reporting Items for Systematic Reviews and Meta-Analysis) [28]. The protocol was registered on the PROSPERO platform (International Prospective Register of Systematic Reviews) with registration number CRD42022340782 before proceeding with item selection.

### 2.2. Eligibility Criteria, Sources of Information, Risk of Bias, Research, and Selection

The search of bibliographic sources from the articles and reports was directed toward all randomized, non-randomized, prospective, and retrospective clinical studies that investigated the role of miR-197 in HNSCC, with a clear correlation with prognostic survival indices and the expression of miR-197.

The PICO question was as follows: Is there a correlation between the prognostic indices of survival: OS—overall survival, PFS—progression—free survival, RFS—relapse-free survival, DFS—disease-free survival, CSS—cancer-specific survival, and an altered expression of miR-197 in HNSCC patients?

The different points studied were: (P) participants (patients with HNSCC), (I) intervention (impaired expression of miR-197 in HNSCC), (C) control (patients with HNSCC who have low expression of miR-197), and (O) outcome (difference in survival prognosis between patients with low and high miR-197 expression in HNSCC).

The exclusion criteria for the systematic review were: (1) all studies that did not report data on tissue miR-196 expression in HHSCC or its histological subtypes, such as LSCC, OPSCC, HSCC, and OSCC; (2) studies published in a language other than English; (3) all studies that did not report prognostic indices of survival; and (4) all literature reviews (considered bibliographic sources only), case reports, and case series.

We considered articles on miR-197 that presented results for relative risk (RR), hazard ratio (HR), Cox regression, or Kaplan–Meier survival curves on prognostic survival indices for HNSCC as potentially eligible for meta-analysis. The review of the studies involved two reviewers (M.D., and S.C.). The research and selection phases of the articles were divided into the following five phases:

1. Choice and decision on the inclusion and exclusion criteria to be adopted, the data banks and the keywords to be used, and the period of time in which to conduct the research;
2. Research and selection of studies performed independently;
3. Removal of overlaps using reference management software such as EndNote 8.0;
4. Choice of studies to include;
5. Comparison of the included studies and resolution of any conflicts between the two reviewers with the help, if necessary, of a third and fourth reviewers (G.T. and A.B.).

The keywords used were miR-197, miR-197 AND HNSCC, LSCC AND miR-197, OSCC AND miR-197, MicroRna AND HNSCC.

The research was conducted on five databases—Science Direct, SCOPUS, EBSCO, Web of Science, and PubMed—and one registry: the Cochrane Central Trial. In addition, Google Scholar (keywords miR-197), gray literature sources such as Open Gray (keywords miR-197), and references from previous systematic reviews on miR and HNSCC were consulted.

In particular, below are all the keywords used in the PubMed search: miR-197 OR microRNA-197, sorted by “Most Recent” plus “miR-197” [All Fields] OR “microRNA-197” [All Fields].

Search: (miR-197 OR microRNA-197) AND HNSCC Sort by: Most Recent (“mir-197” [All Fields] OR “microRNA-197” [All Fields]) AND (“HNSCC” [All Fields] OR “squamous cell carcinoma of head and neck” [MeSH Terms] OR (“squamous” [All Fields] AND “cell” [All Fields] AND “carcinoma” [All Fields] AND “head” [All Fields] AND “neck” [All Fields]) OR “squamous cell carcinoma of head and neck” [All Fields] OR “HNSCC” [All Fields]). Translations HNSCC: “hnsccs” [All Fields] OR “squamous cell carcinoma of head and neck” [MeSH Terms] OR (“squamous” [All Fields] AND “cell” [All Fields] AND “carcinoma” [All Fields] AND “head” [All Fields] AND “neck” [All Fields]) OR “squamous cell carcinoma of head and neck” [All Fields] OR “hnscc” [All Fields].

The search of the literature was completed on 12 June 2022, and a final update of the research was carried out on 3 August 2022.

The data to be extracted from the included articles were decided in advance by the two reviewers and concerned the first author of the study, the date of publication, the country where the research was conducted, the type of squamous cell carcinoma, the number of patients, the clinical characteristics of patients and tumors included in the studies (age, gender, smoking, HPV positive, follow-up, grading, staging), the miRs studied, the value or type of cut-off between the low and high expression of miR-197, RR, and HR values for various prognostic survival indices.

Furthermore, if only the Kaplan–Meier survival curves were present, the hazard ratio was calculated using the Tierney method by extrapolating the data from the curve with Engauge Digitizer 4.1 (open source, non-commercial project, https://markummitchell.github.io/engauge-digitizer/, accessed on 1 August 2022) and reported in a special Excel spreadsheet, available online as supplementary material to the publication by Tierney et al. [29].

In addition, the TGCA (The Cancer Genome Atlas) database containing a cohort of patients with HNSCC was consulted to extract the HR values regarding the prognostic indices linked to the expression of miR-197 [30,31].

The risk of bias in individual studies was evaluated by two authors (MD and SC), as a tool for assessment parameters, derived from the reporting recommendations for prognostic studies of markers (REMARK). Studies with a high risk of bias were excluded from the meta-analysis [32,33].

The evaluation of heterogeneity was carried out using the Higgins index (*I*^2^) and Chi^2^. For the meta-analysis, and particularly for the calculation of the pooled HR or RR, the software Reviewer Manager 5.4 (Cochrane Collaboration, Copenhagen, Denmark) was used. The online software GRADE pro-Guideline Development Tool (GRADEpro GDT, Evidence Prime, Hamilton, ON, Canada) was used to assess the quality of the evidence [34].

Trial sequential analysis (TSA) was performed using Stata 13 (StataCorp, College Station, TX, USA), with the implementation of the R 4.2 software and by installing the id-bounds and metacumbounds commands.

## 3. Results

The search for Science Direct, SCOPUS, PubMed, EBSCO, Web of Science, and Cochrane Central Trial returned 1119 bibliographic sources. With the removal of duplicates, 479 potentially eligible articles were obtained, and for 11 articles, only 1 fully complied with the inclusion and exclusion criteria, so the relevant extracted data were included in the meta-analysis. Furthermore, the gray literature analysis (http://www.opengrey.eu, accessed on 1 August 2022, DANS EASY Archive and Google Scholar) and previous systematic reviews did identify any more studies to be included in the meta-analysis.

In addition, a TGCA analysis was performed through the Kaplan–Meier plotter database portal (https://kmplot.com/analysis/, accessed on 1 August 2022), and HR data were extracted.

**Figure 1:**
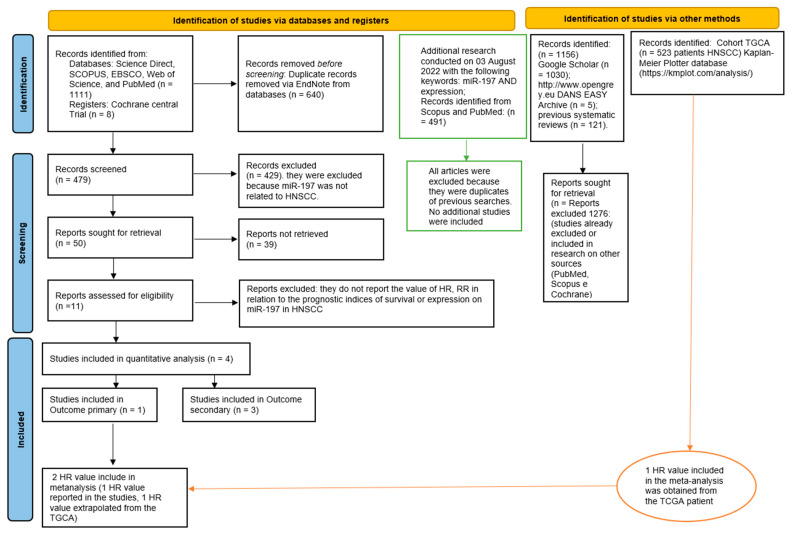
Entire selection and screening procedures are described in the PRISMA flowchart. In total, 2 HR (Hazard Ratio) values were included in the meta-analysis; the first value was taken from the study included in the primary outcome, and the second HR value was obtained from the bioinformatics analysis of the TGCA cohort. After the removal of duplicates, 429 studies were excluded since miR-197 was only mentioned in the text or not related to HNSCC. Subsequently, 39 more studies were excluded because they did not report any miR-197 expression values or prognostic indices of survival. In the green boxes, a further search of articles is shown, which was conducted on August 3, 2022, and did not lead to the inclusion of any additional studies.

The entire procedure for the identification, selection, and inclusion of the studies is indicated in the flowchart of Figure 1.

**Figure 2:**
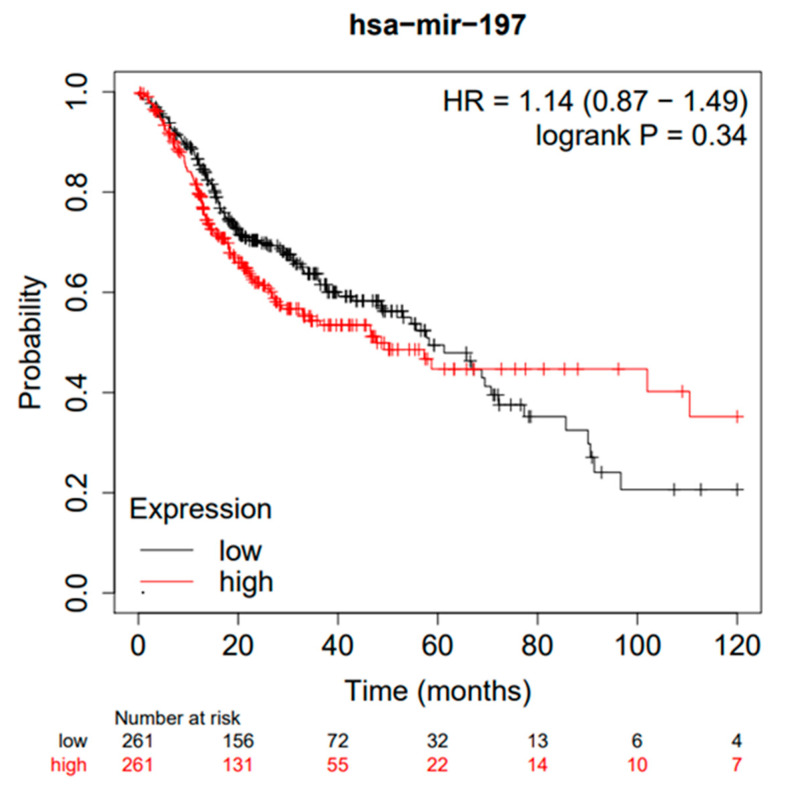
Kaplan–Meier curves according to the miR-197 expression levels for overall survival (OS) of patients with HNSCC (TGCA cohort). Kaplan–Meier curves created by the public database and web application Kaplan–Meier plotter (http://kmplot.com/analysis/, accessed on 1 August 2022).

The analysis of the TGCA cohort, through the Kaplan–Meier plotter database portal (https://kmplot.com/analysis/, accessed on 1 August 2022), on a cohort of 512 HNSCC patients generated the following Kaplan–Meier curves between high and low Mir-197 expressions. For the cut-off value between high and low expression, the median was selected as a parameter for the follow-up period of 120 months (Figure 2). The HR for OS between high and low expressions was found to be 1.14 (0.87–1.49), with a median survival of 58.27 months for the low-expression cohort and 47.67 months for the high-expression cohort (Figure 2).

**Figure 3:**
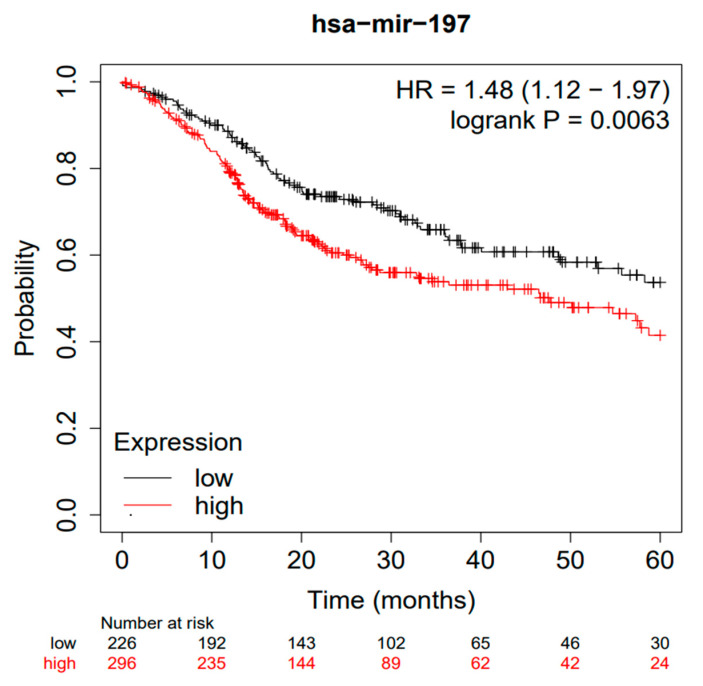
Kaplan–Meier curves according to the miR-197 expression levels for overall survival in patients with HNSCC (TGCA cohort analysis), with a follow-up period of 60 months.

When performing a further analysis after a follow-up period of 60 months, the data became more strongly in favor of a worsening prognosis in the presence of a high expression of miR-197, with an HR of 1.48 (1.12–1.97) (Figure 3).

Four studies were included in the systematic review, but only one had the correct characteristics for inclusion in the meta-analysis as per the main outcome (correlation between prognostic survival indices and altered expression of miR-197 in HNSCC patients): Ahn et al. 2017 [35], as reported in Table 1.

The other three studies, while reporting the miR-197 expression value in HNSCC patients, did not have HR values, and were not included in the main outcome. Moreover, the data were extracted and considered secondary outcomes and are reported in Table 2: Prasad et al. [36], Scapoli et al. [37], and Yang et al. [38].

A total of seven articles were excluded because the data referred to cell lines. The results of the study by Yang et al. included information concerning the presence of an altered expression of miR-197 in saliva, correlating this with the progression of oral precancerous lesions. This pathophysiological aspect of miR-197 may be crucial in the screening of oral carcinoma lesions [38].

Similarly, Scapoli et al. identified how the presence of miR-197 is physiopathologically altered in carcinomatous tissues, noting that the latter may be a biomarker of the presence of the neoplastic lesion [37]. These data are in agreement with the data of Prassad, which demonstrates that miR-197 expression values are physiologically altered in tumors and the oral lichen Planus by comparing them to findings in contralateral oral tissues [36].

These results show that, from a pathophysiological point of view, there is an altered expression of miR-197 in pathological tumor tissues, and, as Ahn et al. demonstrated, this can be correlated with a different expression of PD-L1 and poor OS [35].

The risk of bias was assessed through parameters derived from REMARK. Based on the REMARK guidelines, a score from 0 to 3 was considered for each factor (Table 3). The different points addressed in the REMARK scale were as follows: (1) sample (describe type of biological material evaluated, including control samples and methods of preservation and storage); (2) clinical data (basic clinical data such as age, gender, clinical stage and histopathologic grade are provided); (3) marker quantification (specify the assay method used and provide or reference a detailed protocol, including specific reagents or kits used, quality control procedures, reproducibility assessments, quantification methods, plus scoring and reporting protocols); (4) prognostication (the analyses of survival endpoints are defined); (5) statistics (well-described cutoff point to divide the cases into risk groups, the estimated effect describing the relationship between the evaluated biomarker and the outcome was provided, and an adequate statistical analysis was performed to adjust the estimation of the biomarker effect for known prognostic factors); (6) classical prognostic factors (the prognostic value of the classical prognostic factors and the relationship between the evaluated biomarker/s and classical prognostic factors were reported).

This rating scale has been widely used in other publications on prognostic biomarkers, such as: Almangush et al. [39] and Troiano et al. [40].

Specifically, the study by Ahn et al. [35] used a score of 2 out of 3 because the study sample reported the sample preservation method (formalin-fixed, paraffin-embedded tissue specimens) without adding further references. The clinical data were assigned a score of 2 because they provide clinical–pathological information on the tumor and patient gender and age data but not on the presence of comorbidities. Marker quantification was given a score of 3, and the methodologies and protocols performed are well described in the Materials and Methods section. A score of 3 was assigned to the statistics. Finally, routine prognostic factors were assigned a score of 2 because only DFS and OS were taken into consideration.

**Figure 4:**
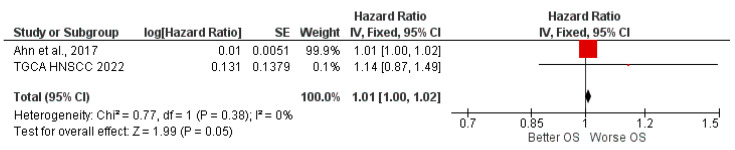
Forest plot of the fixed-effects model of the meta-analysis: OS, HR = 1.01, 95% CI: [1.00, 1.02]; df = degrees of freedom; I^2^ = Higgins heterogeneity index, I^2^ < 50%, heterogeneity irrelevant; I^2^ > 75%, significant heterogeneity; C.I. = confidence intervals; P = *p*-value; SE = standard error. The graph for each study shows the lead author and the date of publication, the hazard ratio (HR) with confidence intervals, the log HR standard error, and the weight of each study expressed as a percentage. The final value is expressed in bold with the relative confidence intervals. The black line shows the position of the average value, and the rhombus in light black shows the measure of the average effect [35].

By obtaining the data from the studies and the bioinformatics analysis of the TGCA relating to HR for OS between high and low miR-197 expression, a meta-analysis of the data was performed; the heterogeneity between the included data was low, with a Chi^2^, df = 1 (*p* = 0.38) equal to 0.77, and an *I*^2^ equal to 0%, which was applied to a fixed effects model. The results of the meta-analysis reported an aggregate HR for OS between high and low miR-197 expressions of 1.01, with relative confidence intervals [1.01 1.02], and the test for the overall effect was Z = 1.99 (*p* = 0.05) (Figure 4). The results of the two single studies (Ahn et al. and TGCA HNSCC cohort analysis) are slightly in favor of prognostic indices of worsened survival in the presence of high miR-197 expression, although they are not statistically significant.

There is a strong limit to the meta-analysis determined by the inclusion of only two HR data, of which those derived from the study by Ahn et al. have an excessively high weight due to the presence of a very narrow confidence interval, which is not observed in the bioinformatic analysis of the TGCA cohort.

For the meta-analysis, and particularly for the calculation of the pooled HR between high and low miR197 expression, the software Reviewer Manager 5.4 (Cochrane Collaboration, Copenhagen, Denmark) was used. In particular, the GRADE pro-Guideline Development Tool online software (GRADEpro GDT, Evidence Prime, Hamilton, ON, Canada) was used to assess the quality of the evidence, following the recommendations described in Chapter 12, Section 2 of the Cochrane Handbook for Systematic Reviews of Interventions [34]. To generate the summary table of results for the primary outcome (Table 4), the five criteria used were: study limitations, consistency of effect, imprecision, indirectness, and publication bias.

**Figure 5:**
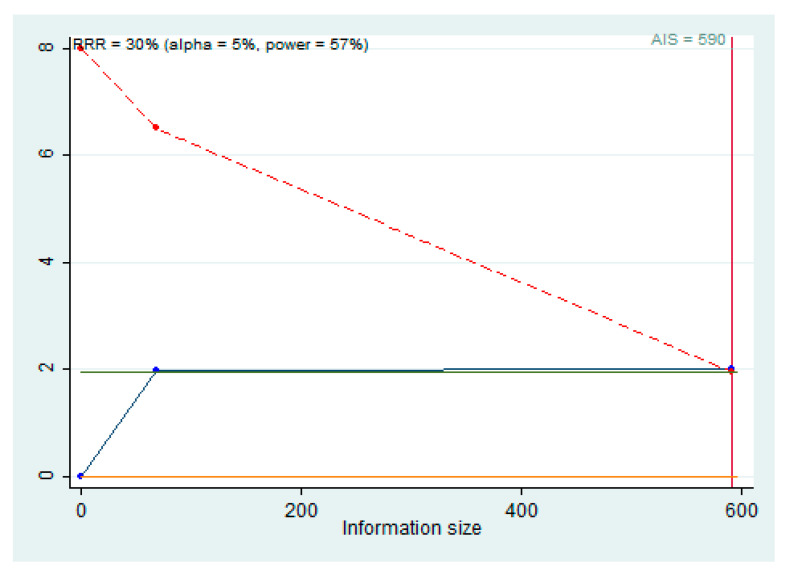
APIS, light green line (Z = 1.98); dashed red line (monitoring boundary); blue line (cumulative z curve); red line (sample size).

**Figure 6:**
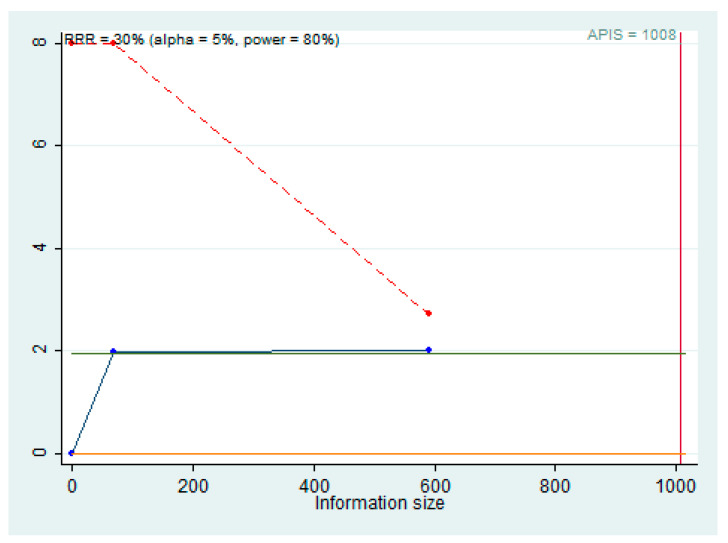
APIS, light green line (Z = 1.98); dashed red line (monitoring boundary); blue line (cumulative z curve); red line (sample size).

The results suggest that the quality of the evidence was low. A trial sequential analysis (TSA) was performed to evaluate the strength of the results of the meta-analysis and to adjust the results to avoid type I and II errors. The program used was Stata 13 (StataCorp, College Station, TX, USA), with the integration of the R 4.2 software through the metacumbounds commands, as described by Miladinovic et al. [41]. The O’Brien–Fleming spending function was used by applying fixed effects. The AIS (accrued information size) (Figure 5), and subsequently APIS (a priori information size) (Figure 5) commands were used by the Dialog BOX to determine the optimal sample size and for the power of the results assuming an RRR (reduction risk relative) of 30%, an alpha value equal to 5% (type 1 error), and a beta value of 20% (type 2 error) (Figure 6).

The TSA curve, crossing line Z = 1.98, provides a significant result but creates a spurious effect because the z curve does not cross the monitoring boundary. This could be a false positive result.

The APIS graph shows that for an RRR of 30%, alpha 5%, and power of 80%, the number of optimal patients is 1008.

## 4. Discussion

We conducted a systematic review of the literature, investigating with a meta-analysis, in addition to TSA, the possible role of miR-197 expression in HNSCC. This review represents the first systematic review to investigate the role of miR-197 in HNSCC. A bioinformatic analysis was conducted on the data present in the TGCA through the Kaplan–Meier plotter database portal (https://kmplot.com/analysis/, accessed on 1 August 2022) by integrating the results with the data present in the literature through a meta-analysis of the data.

In total, 580 patients were included in the meta-analysis, of which 512 were from the TGCA cohort and HNSCC, and 68 were from the study by Ahn et al., the latter of whom were specifically OSCC [35].

The data extracted for TGCA concerned the HR for OS between the high and low expressions of miR-197, while for the Ahn study, in addition to OS, DFS was also available, for which it was not considered appropriate to perform a meta-analysis due to the lack of included data [35]. On the other hand, from the analysis performed by the Kaplan–Meier plotter, it is interesting to see how, upon reducing the follow-up period to 60 months, the OS tended to be higher in the group of patients presenting a high expression of miR-197, with an HR of 1.47. Consequently, these data suggest that miR-197 can serve as a putative biomarker for short-term (5 years) survival (Figure 3).

Aggregated HR for OS reported a result of 1.01, and it is evident that there is no predictive effect on prognosis by aggregating the results of the two studies. An analysis of the HR of the TGCA cohort analysis reveals a value of 1.14 with wider confidence intervals than the study by Ahn et al., which also found an increase in the weight of the meta-analysis for the TGCA cohort. However, when the confidence intervals are not taken into account, the two data in the meta-analysis are aligned in a position of worsening prognosis with an improvement in the presence of a high expression [35].

The TSA provides further information about the power of the results obtained from the meta-analysis. In fact, the results shown by the AIS and APIS graphs are slightly in favor of a worsening in the course of the high expression of miR-197 exceeding the regression z-line (Z = 1.98) but not the monitoring boundary line. Moreover, it can be seen from the APIS graph that the ideal number of patients to be recruited in the studies is 1008; therefore, the results obtained could be false-positive results, and only the implementation of other studies could provide an outcome with adequate statistical power.

The data included in the secondary outcome, in which we investigate the levels of expression of miR-197 in HNSCC patients, would seem to indicate a different outcome. All the included studies concur that in the presence of malignant tissue transformation, there is a downregulation of miR-197. In fact, clinical prognostic characteristics cannot ignore the pathophysiological knowledge of the function of miR-197, which remains somewhat unknown. From an analysis of the literature, we can identify roles that have yet to be defined in angiogenesis, cell proliferation, apoptosis, tumor invasion, and metastasis. These aspects can be decisive and crucial in carcinogenesis and can potentially have an impact on tumor therapy and prognosis.

Angiogenesis is a fundamental process in the growth and progression of tumors, and miR-197 promotes angiogenesis in cancer by increasing the VEGF-A/VEGFR2 pathway by binding to the CYP4Z2P and CYP4Z1 genes [42], and by activating the PI3K/pathways AKT and ERK1/2 [26]. All processes involved in tumor neo-angiogenesis, and the mechanisms of action in tumor cell proliferation, involve the ACVR1 and TSPAN3 genes, which are target genes for miR-197 and the IGFBP5 receptor [43].

The mechanism of action against apoptosis occurs through interaction with genes such as p53 [24], NOXA [24], BMF, STAT6, and FOXJ-2 [22];, as well the mechanism of action against p53. It is believed that p53 is inhibited by miR-197, and the consequent level of apoptosis decreases. In addition, STAT6 silencing improves apoptosis by inhibiting miR-197, which negatively regulates FOXJ-2 by altering the expression of cholesterol biosynthesis. Furthermore, miR-197 seems to affect the expressions of caspase-9, caspase-3, MCL-1 [44], RARP, BID, BAD, BAX, Bcl-2, and Bcl-XL, leading to apoptosis [45]. Moreover, miR-197 seems to play a role in tissue metastasis and invasion through direct action in CD82/KAI-1 [46] and p120 catenin [47], as well as indirect action in the Rac-1 and ROCK genes [48].

In particular, evidence suggests that CD82/KAI-1 is a metastasis suppressor factor in several cancers, and miR-197 seems to indirectly activate two genes that induce cellular metastases, ROCK, and Rac 1 [48], through direct action in CD82/KAI-1. Additionally, several tumor suppressor genes involved in tumor growth metastatic invasion, such as CTNNAI, IKZFI, or EBF3, can be identified as new targets of miR-197 [49].

The overexpression of miR-197 downregulates p120 catenin, which, binding to the E-cadherin promoter, influences the epithelial–mesenchymal transition (EMT) [50], an important process for tumor invasion and metastasis [47].

This information on the oncosuppressive role of miR-197 overexpression is reflected in clinical studies conducted on HNSCC. In fact, according to the saliva study conducted by Yang et al., which was carried out on the progression and malignant transformation of leukoplakia lesions, miR-197 appears to be overexpressed in patients with non-progressing leukoplakia, confirming its tumor suppressor role (miR 10b, miR-145, miR-99b, miR-708, miR -181c, miR-30e, miR-660) [38]. Based on these data, salivary biomarkers, including miR-197, represent a promising method for measuring the risk of malignant transformation of precancerous lesions, even if biopsy remains the gold standard for the diagnosis of oral cancer.

These data were also confirmed by Prasad et al., who compared OSCC samples of lichen, dysplasia, and histologically normal epithelium [36], and in a retrospective study of 53 patients with OSCC conducted by Scapoli et al., in which under-expressed miR-197 was reported as statistically significant, along with other miRs (miR-520h, miR-378, miR-135b, miR-224, and miR-34a) [37]. Moreover, Ahn et al. also performed an immunohistochemical study on OSCC samples, demonstrating that miR-197 expression is inversely correlated with PD-L1 expression and that a high expression of miR-197 was weakly associated with poor OS (*p* = 0.033) [35].

The clinical studies’ outcomes for the expression of miR-197 are in line with the results obtained for cell lines (not included in the current systematic). In fact, miR-197 was downregulated in the study by Dai et al., together with two other miRs (miR-100, miR-130), in head and neck squamous cell carcinoma cell lines UMSCC-1 [51], which confirms the data of Kozaki et al. [52], while Jiang et al. demonstrated that exosomes loaded with miR-197-3p inhibit proliferation and neoplastic migration in nasopharyngeal carcinoma (NPC) cell lines [53].

Although the present meta-analysis was performed following the Cochrane handbook indications, and reported using the PRISMA guidelines, we found some limitations. A strong limitation of this meta-analysis is the lack of data included, despite the fact that six databases (Science Direct, SCOPUS, PubMed, EBSCO, Web of Science, and Cochrane Central Trial) and Google Scholar were used, with further research on gray literature and conference abstracts conducted to address publication bias. Another limitation of the meta-analysis is that data were extracted from published results rather than individual patients’ records. These data could reflect a wide variation in miR-197 expression in the HNSCC population, limiting their use as prognostic biomarkers in clinical practice. This topic should be evaluated in further studies with different designs.

## 5. Conclusions

In conclusion, from the literature data included in the meta-analysis, TSA, and bioinformatics analysis data, miR-197 does not currently represent a valid prognostic biomarker for HNSCC, although the data provided by the Kaplan–Meier plotter suggest that miR-197 can serve as a putative biomarker for short-term (5 years) survival. Nevertheless, further research is necessary given the low number of current studies, especially in light of the data reported in the international scientific literature on the expression of miR-197, which clearly highlights the ongoing downregulation of HNSCC. Consequently, exhaustive investigations of miRNA—for instance, investigations regarding the intercommunication among miRNAs and between miRNAs and other genes, the altered protein expression induced by miRNAs, and site-specific miRNA expression profiling—are prerequisites for future clinical trials of therapeutic applications. Furthermore, extensive research is still required before the concrete function and mechanisms of HNSCC can be fully elucidated.

## Figures and Tables

**Table 1:** The data extracted for the study included in the meta-analysis.

Lead Author, Data	Country	Study Design	Age	Number of Patients (Male, Female); Staging ^1^ (I-II, III-IV).	Smoking History (Y, N), Alcohol History (Y, N)	Follow-Up Max	Tumor Type/	Cut-Off	miR	HR miR-197 High or Low Expression (OS, DFS)
Ahn et al., 2017 [35]	Korea	retrospective	57.7 years (range = 23–84 years)	68 (45, 23) Staging (35, 33)	\	44.3 months (range, 2.1–122.0 months)	OSCC 68, formalin-fixed, paraffin-embedded	Median	miR.197	DFS: HR 1.01 (1.00–1.02) *p* = 0.089 OS:HR 1.01 (1.00–1.02) *p* = 0.033

^1^ staging classification by the seventh edition of the American Joint Committee on Cancer (AJCC), \ Data not available.

**Table 2:** The data extracted for the study included in the secondary outcomes.

First Author	Country	miR	Patient	Age (Range)	Smoker	Grading (G1,G2,G3)	Follow-Up (Range)	Type Sample	Type HNSCC	Expression of miR-197 (Fold Change or SAM Score)
Yang et al. 2013 [38]	China	miR-10b-5p, miR-99a-5p, miR-99b-5p, miR-145-5p, miR-100-5p, miR-125b-5p, miR-181, miR-181c, miR-197-3p, miR-331-3p, miR-15a-5p, miR-708, miR-150-5p, miR-30e-3p, miR-30a-3p, miR-21, let-7a-5p, miR-335-5p, miR-144, miR-25-3p, miR-19a-3p, miR-660-5p, miR-140-5p, miR-590-5p, miR-921.	15 (8 male 7 female)	45–71 years	(5 never, 3 former, 7 current)	\	24–40 months	saliva	OSCC, oral premalignant lesions (OPL), high grade dysplasia (HGD).	16.89↓ *p* = 0.042 (progressing leucoplakias e non-progressing leucoplakias)
Scapoli et al. 2010 [37]	Italy	miR489, miR-129, miR-23a, miR-214, miR-23b, miR-92, miR-25, miR-210, miR-212, miR-515, miR-146b, miR-21, miR-338, miR-520h, miR-197, miR-378, miR-135b, miR224, miR-34a, mir-155, let-7i, mir-146a	15 (12 male, 3 female)	51–93 years	\	Grading (G1 n = 1, G2 n = 6, G3 = 8)	12–26 months	Tissue frozen	OSCC	Microarray SAM score −3.04
Prasad et al. 2017 [36]	Australia	miR-155, miR-24, miR-26b, miR-21, miR-127, miR-197	50?	\	\		\	formalin fixed, paraffin embedded	20 OSCC, 20 histologically normal epithelium (HNE), 10 with mild dysplasia, 10 with moderate or severe dysplasia, and 10 OLP	4.40 between OLP e HNE

\ Data not available.

**Table 3:** Assessment of the risk of bias within the studies. Scores 8 to 10 = low quality, 11 to 14 = medium quality, and 15 to 18 = high quality.

Lead Author, Data	Sample	Clinical Data	Marker Quantification	Prognostication	Statistics	Classical Prognostic Factors	Score
Ahn et al. 2017 [35]	2	2	3	3	3	2	15

**Table 4:** Evaluation of GRADEpro GDT; CI, confidence interval; HR, hazard ratio, ⨁⨁◯◯ Low.

Certainty Assessment							N° of Patients		Effect		Certainty
N° of Studies	Study Design	Risk of Bias	Inconsistency	Indirectness	Imprecision	Other Considerations	miR-197 High	mir-197 low	Relative (95% CI)	Absolute (95% CI)	
2	observational studies	not serious	not serious	not serious	not serious	publication bias strongly suggested thatall plausible residual confounding would imply a spurious effect, while no effect was observed	290	290	HR 1.01 (1.00 to 1.02)	1 fewer per 1.000 (from 1 fewer to 1 fewer)	⨁⨁◯◯ Low

## Data Availability

Not applicable.
